# Supplementary material for: Immunogenic Cell Death Photothermally Mediated by Erythrocyte Membrane-Coated Magnetofluorescent Nanocarriers Improves Survival in Sarcoma Model
Source: Pharmaceutics. 2023 Mar 14;15(3):943. doi: 10.3390/pharmaceutics15030943 (PMC10051374; doi:10.3390/pharmaceutics15030943)
Supplement: Supplementary file 1 [file pharmaceutics-15-00943-s001.zip › pharmaceutics-2204173-supplementary.pdf]

Immunogenic Cell Death Photothermally Mediated by Erythrocyte Membrane-Coated Magnetofluorescent Nanocarriers Improves Survival in Sarcoma Model

Ailton Antonio Sousa-Junior [1,2], Francyelli Mello-Andrade [3,4], João Victor Ribeiro Rocha [1], Tácio Gonçalves Hayasaki [2], Juliana Santana de Curcio [3], Lívia do Carmo Silva [3], Ricardo Costa de Santana [1], Eliana Martins Lima [2,5], Cléver Gomes Cardoso [3], Elisângela de Paula Silveira-Lacerda [3], Sebastião Antonio Mendanha [1,2,5] and Andris Figueiroa Bakuzis [1,5,*]

[1] Institute of Physics, Federal University of Goias, Goiania 74690-900, GO, Brazil
[2] FarmaTec, School of Pharmacy, Federal University of Goias, Goiania 74690-631, GO, Brazil
[3] Biological Sciences Institute, Federal University of Goias, Goiania 74045-155, GO, Brazil
[4] Federal Institute of Education, Science and Technology of Goias, Goiania 74055-110, GO, Brazil
[5] CNanoMed, Federal University of Goias, Goiania 74690-631, GO, Brazil
* Correspondence: bakuzis@ufg.br

Supplementary Information

TEM images and EDS spectra

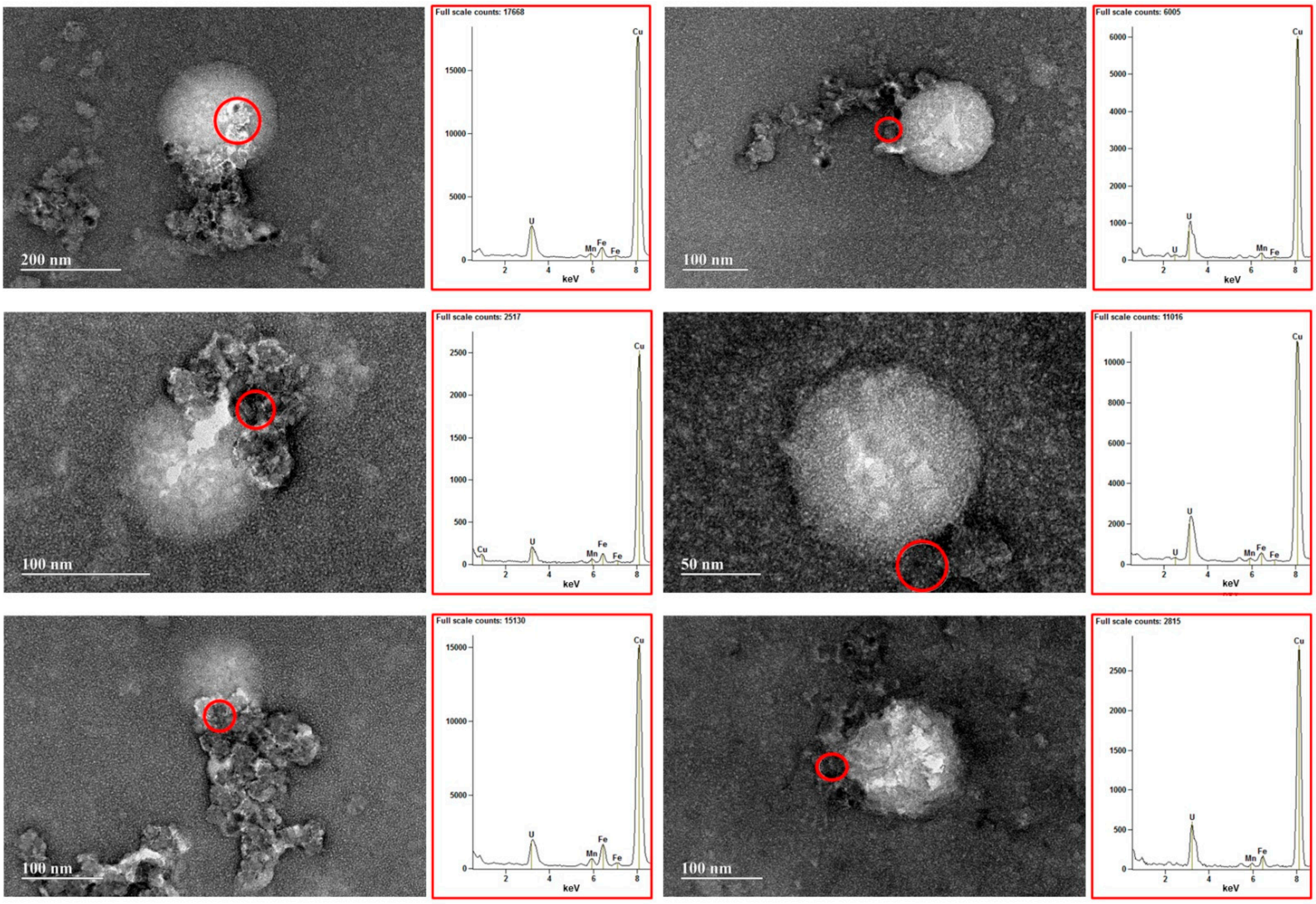

Figure S1 – Representative RBCm-IR-Mn TEM images and corresponding EDS (energy dispersive spectroscopy) spectra

NTA size distributions

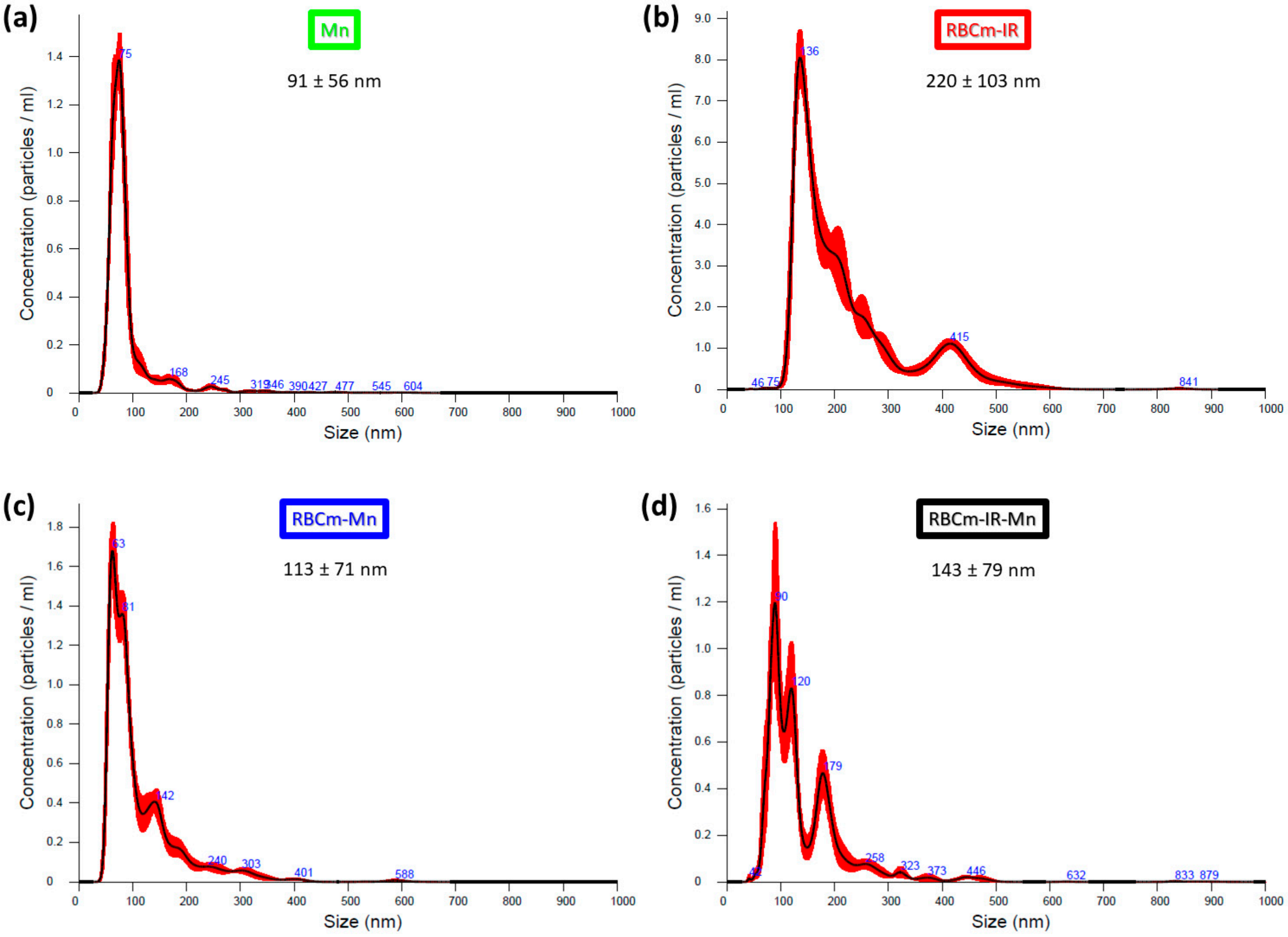

Figure S2 – (a) Mn-ferrite nanoparticles. **(b)** RBCm-IR control sample.
(c) RBCm-Mn control sample. **(d)** RBCm-IR-Mn nanocarrier. Average diameters are reported as mean ± SD.

RBCm-IR photostability

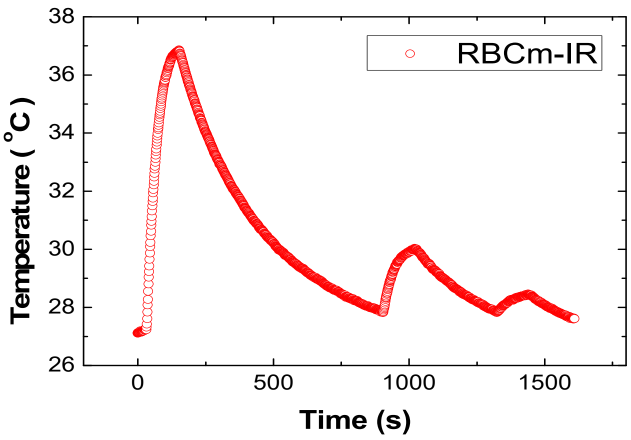

Figure S3 – RBCm-IR shows photobleaching under sequential PTT cycles.

RBCm-IR-Mn cooling time fit

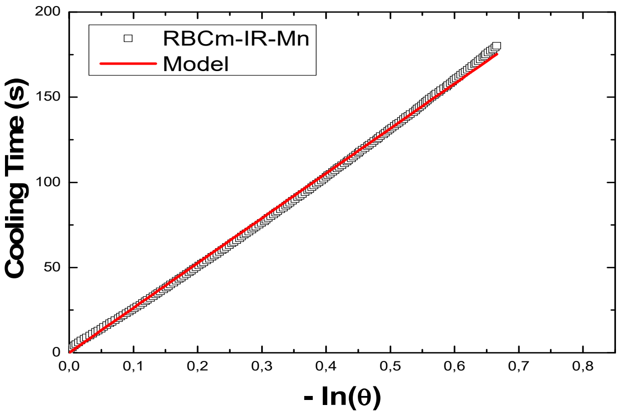

Figure S4 – Linear fit corresponding to the cooling time of RBCm-IR-Mn (step for PCE determination).

Cell viability study without PTT

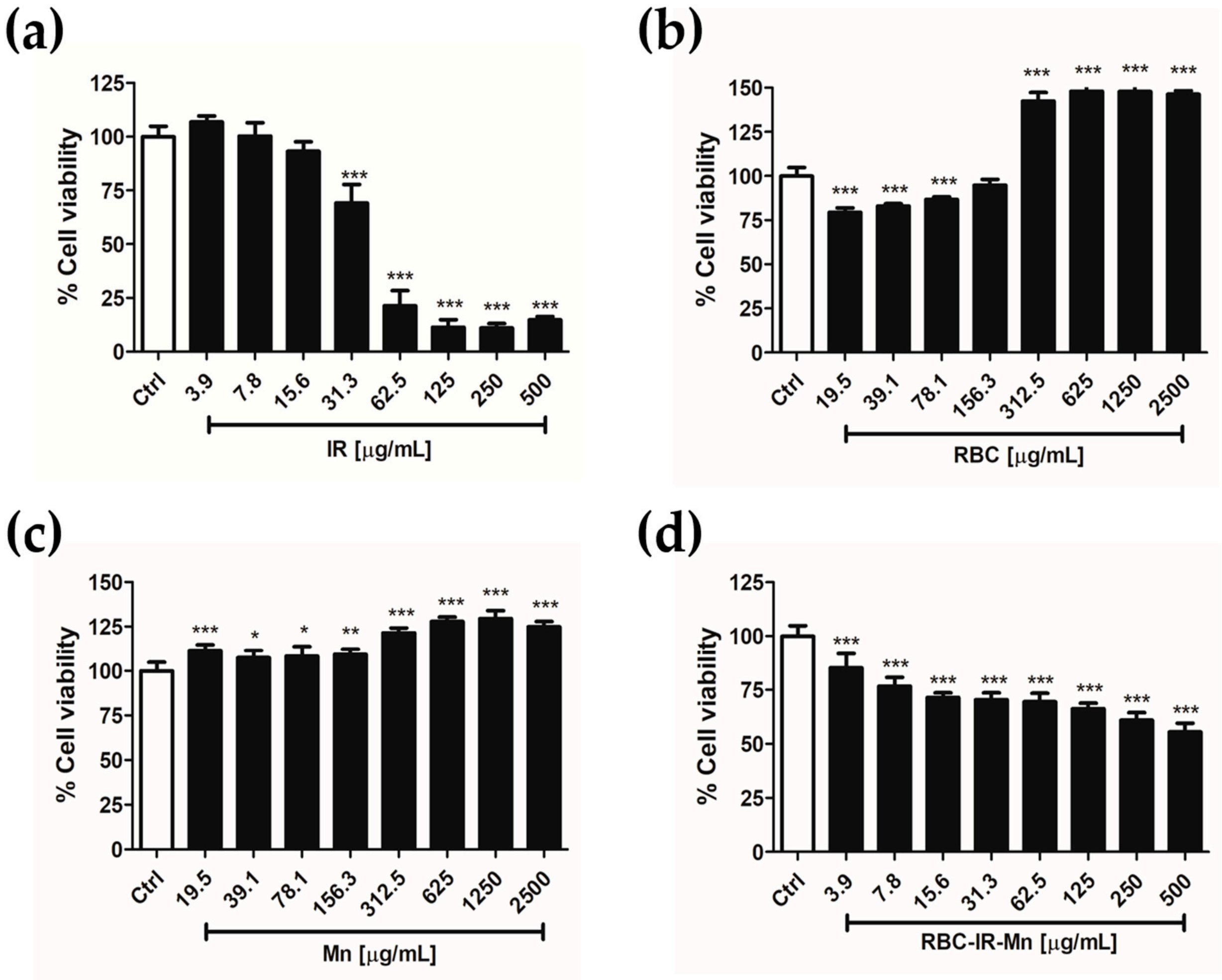

Figure S5 – Cell viability study without laser irradiation. MTT study as function of the concentration of **(a)** free IR-780 concentration; **(b)** erythrocyte membrane vesicles (without IR-780 and without magnetic nanoparticles); **(c)** Mn-ferrite nanoparticles; and **(d)** RBCm-IR-Mn nanocarriers.

PTT for nonspecific laser heating

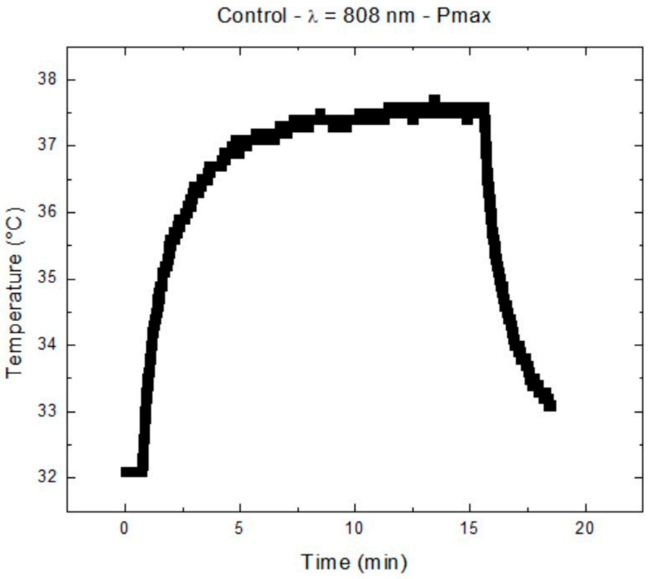

Figure S6 – Control tumor-bearing animal submitted to PTT for the determination of nonspecific heating.

Tumor Evolution and PTT treatments for animal GM-SM

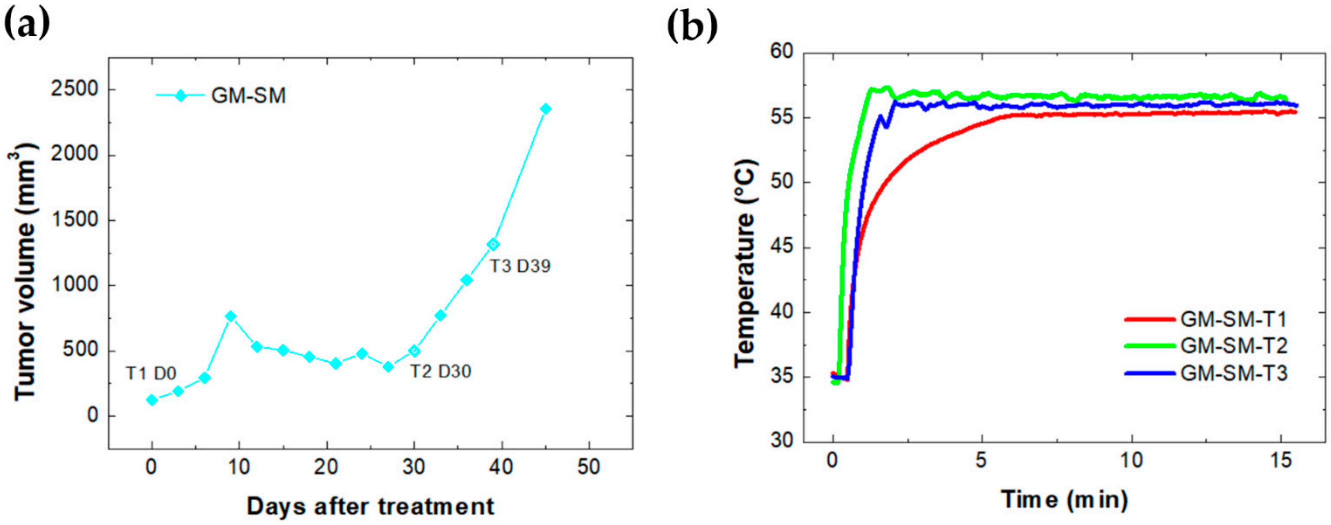

Figure S7 – (a) Tumor volume evolution and **(b)** temperature profile for PTT treatments performed for animal GM-SM

In vivo **preclinical PTT study – Average profiles**

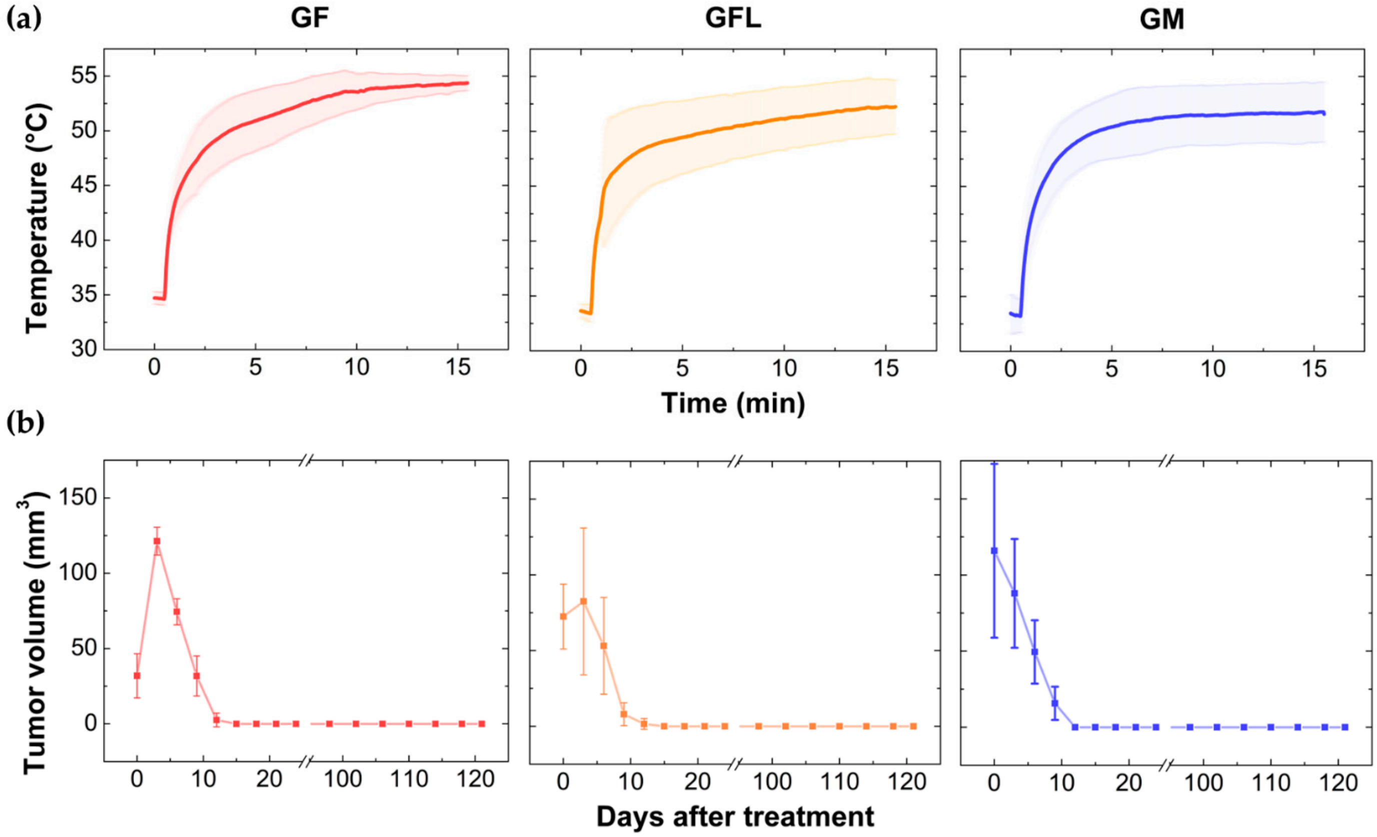

Figure S8 – (a) Mean PTT temperature profiles and **(b)** Mean tumor volume evolution for animals in groups GF ($n = 4$), GFL ($n = 5$), and GM ($n = 4$). Datapoints are expressed as mean ± SD.
